# Targeting Bone Metabolism in Patients with Advanced Prostate Cancer: Current Options and Controversies

**DOI:** 10.1155/2015/838202

**Published:** 2015-01-31

**Authors:** Tilman Todenhöfer, Arnulf Stenzl, Lorenz C. Hofbauer, Tilman D. Rachner

**Affiliations:** ^1^Department of Urology, University Hospital, Hoppe-Seyler-Straße 3, 72076 Tübingen, Germany; ^2^Vancouver Prostate Centre, University of British Columbia, 2660 Oak Street, Vancouver, BC, Canada V6H 3Z6; ^3^Department of Medicine III, Technische Universität Dresden Medical Center, Fetscherstraße 74, 01307 Dresden, Germany

## Abstract

Maintaining bone health remains a clinical challenge in patients with prostate cancer (PC) who are at risk of developing metastatic bone disease and increased bone loss due to hormone ablation therapy. In patients with cancer-treatment induced bone loss (CTIBL), antiresorptive agents have been shown to improve bone mineral density (BMD) and to reduce the risk of fractures. For patients with bone metastases, both zoledronic acid and denosumab delay skeletal related events (SREs) in the castration resistant stage of disease. Novel agents targeting the Wnt inhibitors dickkopf-1 and sclerostin are currently under investigation for the treatment of osteoporosis and malignant bone disease. New antineoplastic drugs such as abiraterone, enzalutamide, and Radium-223 are capable of further delaying SREs in patients with advanced PC. The benefit of antiresorptive treatment for patients with castration sensitive PC appears to be limited. Recent trials on the use of zoledronic acid for the prevention of bone metastases failed to be successful, whereas denosumab delayed the occurrence of bone metastases by a median of 4.1 months. Currently, the use of antiresorptive drugs to prevent bone metastases still remains a field of controversies and further trials are needed to identify patient subgroups that may profit from early therapy.

## 1. Background

In patients with prostate cancer, tumor- and treatment-related changes in bone metabolism have a significant impact on morbidity and cancer-related outcome. Both metastatic bone disease and cancer-treatment induced bone loss (CTIBL) impair bone stability and increase the risk of fractures leading to immobility, pain, and significant decrease in quality of life. The hospitalization associated with the occurrence of skeletal related events is associated with a high health-economic burden, and previous studies have shown that skeletal related events double the annual treatment-related costs in patients with metastatic prostate cancer [1]. Therefore, current treatment concepts in prostate cancer have a special focus on the prevention of fractures and other skeletal related events [2]. The number of treatments available providing significant benefits for patients with bone metastases has increased considerably in the last 5 years [3]. However, there are still controversies on which patients actually should receive bone-targeted treatments and whether some bone targeted treatments delay or prevent the occurrence of bone metastasis in patients with earlier stages of prostate cancer. Moreover, there is still a discrepancy between guidelines and clinical practice regarding the management of CTIBL, indicating that the potential consequences of increased loss of bone mineral density (BMD) are still underestimated by many urologists [4]. Both vitamin D supplementation and antiresorptive agents provide effective measures for treatment of CTIBL. The current review aims to discuss current concepts in the pathophysiology of cancer-associated changes in bone metabolism and current trends in the treatment of cancer-related bone disease in prostate cancer patients.

## 2. Methods

For the present review, a PubMed search for articles published between January 1, 2000, and November 22, 2014, was performed. Articles with high relevance for the topic published before January 1, 2000, were also included. The search included the terms “prostate cancer,” “bone metastase,” “skeletal related events,” “cancer-treatment induced bone loss,” “androgen deprivation therapy,” “male osteoporosis,” “bone mineral density,” “denosumab,” “zoledronic acid,” and “bisphosphonates.”

## 3. Pathophysiology of CTIBL-Induced Osteoporosis

Although the presence of androgens does not correlate with the risk of developing prostate cancer, approximately 80% of prostate cancers are initially sensitive to androgens and respond to hormone deprivation treatments [5]. Different agents are available to suppress androgen production or receptor signaling in prostate cancer. Available options include gonadotropin-releasing hormone agonists and antagonists, androgen receptor antagonists, and 5*α*-reductase enzyme inhibitors, which prevent the conversion of testosterone to the highly active 5*α*-dihydrotestosterone [6].

Male patients undergoing androgen deprivation therapy experience a rapid loss of bone mass and* vice versa* testosterone therapy reduces bone turnover [7]. While these data suggest a direct correlation between androgens and bone metabolism, there is increasing evidence to suggest that, in fact, circulating levels of estrogens are more closely related to bone loss and fracture risk in men than testosterone levels [8]. In a prospective osteoporotic fracture study in men, those with lowest estradiol and testosterone levels had the lowest BMD and most rapid decline in bone mineral density [7].

The importance of estrogen levels in the maintenance of bone homeostasis is well documented from studies of postmenopausal osteoporosis. In addition, the clinical importance of bone loss related to hormone-ablation in breast cancer patients has been highlighted in the past years. Estrogens have direct receptor mediated effects on bone metabolism by modulating osteoblast and osteoclast activity. In addition, indirect effects are mediated by cytokines and growth factors like TGF-*β*, IGF-1, and TNF members. An important pathway regulated by estrogens is the RANKL/RANK/OPG pathway that determines osteoclast activity [6].

An additional influence of testosterone on bone may be its anabolic influence on muscle mass. There are studies to suggest that sarcopenia results in impaired bone microarchitecture. These effects are mediated by mechanical loading and myokines [9]. Therefore, the commonly observed loss in muscle mass under androgen ablation may indirectly modulate bone mass.

## 4. Management of ADT in Prostate Cancer Patients

Baseline therapy for osteoporosis generally consists of supplementation of vitamin D and calcium as well as the advice to increase physical activity. There is no data from randomized controlled trials showing that increased physical activity and exercise reduces the loss of BMD or fracture risk in patients with ADT [10]. There have been studies to suggest that high calcium intake is associated with an increased risk of prostate cancer. However, there is no evidence that modest calcium supplementation influences prostate cancer progression [11]. Mostly, antiresorptive trials using bisphosphonates or denosumab have been conducted under a baseline supplementation of calcium and despite some unclear data, modest levels of physical exercise, as well as maintenance of calcium intake of 1200 mg daily, and vitamin D supplementation are recommended by experts [10].

Bisphosphonates are established agents for the treatment of different forms of osteoporosis. Several studies using different oral and intravenous bisphosphonates have shown favorable effects on BMD in patients undergoing androgen ablation therapy. Treatment with bisphosphonates was associated with a suppression of bone turnover markers and increases in BMD, independent of baseline BMD [10]. A meta-analyses of 15 randomized trials revealed a significant fracture reduction in ADT patients treated with bisphosphonates (risk ratio (RR), 0.80; *P* = 0.005) [12]. The lowest number needed to treat to prevent a fracture data was obtained for patients receiving zoledronic acid (NNT 14.9), while approximately 40 patients needed treatment with an oral bisphosphonate to prevent one fracture (NNT 38.4 for pamidronate and 41.6 for alendronate). Of note, the benefits of bone loss prevention were reached without major gastrointestinal or cardiovascular side effects.

A new option for the treatment of bone loss in men receiving ADT is denosumab. Denosumab is a human fully monoclonal antibody against RANKL, an essential regulator of osteoclast differentiation and activity. The efficacy of denosumab in the setting of ADT was specifically addressed in the HALT trial [11]. In this trial, men with nonmetastatic prostate cancer receiving denosumab had a significant reduction of new vertebral fractures compared to placebo (1.5 versus 3.9%) after 36 months of treatment. Furthermore, BMD significantly increased at the lumbar spine by 5.6% compared to a loss of 1.0 in the control group (*P* < 0.001). In fact, BMD increases were also noted at all other sites investigated, including lumbar spine, total hip, and distal radius. As a result of these data, denosumab was US Food and Drug Administration (FDA) approved for the treatment of men with a high fracture risk undergoing ADT for nonmetastatic prostate cancer.

While a number of antiresorptive agents are available for the treatment of ADT induced bone loss, there is an unmet need for bone anabolic agents. Treatment with PTH (parathyroid hormone) is contraindicated for the treatment of patients with malignancies [13]. A number of anabolic agents are currently under clinical investigation.

These included antibody based approaches against Wnt inhibitors sclerostin and dickkopf-1 (DKK-1). DKK-1 trials are currently underway for a number of bone oncology indications [14]. Trials using a sclerostin antibody have shown efficacy in increasing BMD in patients with postmenopausal osteoporosis [15]. The concept of sclerostin inhibition may be especially promising in patients with prostate cancer, as elevated levels of sclerostin have been reported in med with prostate cancer receiving ADT [16]. On the other hand, as intermittent human parathyroid hormone 1-34 (hPTH) exposure has been shown to enhance the formation of osteoblastic lesions in a mouse model of prostate cancer and this finding has been linked to elevated levels of bone turnover, the safety of other bone anabolic approaches needs to be assessed very carefully when used in this clinical setting [17]. In fact the importance of bone metabolisms has been highlighted by a recent paper showing that bone parameters are strong prognostic factors of OS in CRPC. Bone turnovers have been also demonstrated to be of prognostic relevance in patients with castration-sensitive PC [18]. Bone turnovers have been also demonstrated to be of prognostic relevance in patients with castration-sensitive PC. In a recently published study, patients who responded to ADT with decreased markers of bone turnover in the absence of antiresorptives had significantly improved SRE-free survival compared to those who did not [19].

## 5. Metastasis Associated Changes in Bone Metabolism

In the healthy skeleton, bone turnover is a tightly controlled process regulated by a complex system of hormones and cytokines to assure the maintenance of bone homeostasis. In malignant bone diseases this normal bone turnover is disturbed by a pathological interaction of tumor cells, cells of the bone compartment and immune cells. Depending on the type of cancer, the morphology of bone lesions can vary between predominantly osteolytic lesions and more osteosclerotic lesions. Bone lesions secondary to prostate cancer feature a state of accelerated bone turnover with an abnormal activation of both osteoclasts and osteoblasts. These lesions commonly have a mixed phenotype consisting of both osteolytic and osteosclerotic areas [6].

While the mechanisms of lytic bone disease are relatively well investigated, the pathophysiology of sclerotic bone lesions is less well understood. Compared to lytic lesions, prostate cancer bone metastases cause an additional pathological activation of unstructured osteoblastic bone formation. While the radiographic appearance of these lesions appears dense and suggests stability, the bone is structurally weak and prone to fractures.

The high bone turnover is reflected by elevated levels of markers of bone resorption and formation. Affected patients often have elevated levels of urinary N-telopeptide (NTx) as a marker of collagen degradation by osteoclasts and elevated levels of bone specific alkaline phosphatase (BAP), which reflects heightened bone formation [20].

At the molecular level the progression of sclerotic lesions is determined by the preference of prostate cancer cells to invade the osteoblastic niche in the bone marrow and to regulate several key pathways of osteoblast function [21]. Osteosclerotic lesions are promoted by the release of osteoblast-promoting factors such as BMPs, WNTs, and endothelin-1.

Endothelin-1, best known for its role as a potent vasoconstrictor, promotes osteoblast differentiation and activity in bone. In patients with prostate cancer bone metastases, serum levels of endothelin-1 are elevated [22]. One way of endothelin-1 to regulate osteoblast function is by inhibition of DKK-1, an inhibitor of Wnt-signaling, in marrow stromal cells [23]. Furthermore, preclinical data provides evidence that blockade of the endothelin-1 receptor can prevent the occurrence of osteosclerotic lesions [24]. However, consequent phase III trials evaluating the potential of endothelin receptor antagonists have failed to provide evidence for a significant clinical benefit in patients with prostate cancer [25, 26].

Bone morphogenetic proteins (BMPs) are members of the TGF*β* superfamily that regulate multiple cellular functions and play a key role in skeletal development. BMPs can bind to a number of receptors expressed on mesenchymal stromal cells and regulate osteoblast differentiation and activity [27]. Prostate cancer cells have been shown to express several BMPs like 2, 4, 6, and 7. In addition to its effects on osteoblasts, BMPs have a proangiogenic potential and this may be an additional mechanism by which metastases are promoted. Experimental inhibition of BMP-6 using an antibody based approach successfully blocks the osteoblastic abilities of prostate cancer cells in animal experiments [28]. In addition, modification of the BMP inhibitor noggin changes the osteoanabolic phenotype of certain prostate cancer cells, further supporting the notion that BMPs play an important role in sclerotic bone lesions [29].

Osteonectin is a glycoprotein predominantly expressed by osteoblasts that binds calcium and shows affinity for collagen. Osteonectin is increased at metastatic sites and promotes the migration and invasion of prostate cancer cells [30].

Insulin-like growth factors consist of two ligands (IGF-I and IGF-II) and two receptors. In bone, the IGF system promotes osteoblast activity and bone formation. There is data suggesting that metastasized prostate cancer cells show an increased activity in IGF-signaling in bone compared to other sites and that this activation promotes the formation of sclerotic bone lesions [31].

While there is abundant preclinical and early clinical data to suggest a role of these pathways in the occurrence of bone lesions, none of these have yet translated into approved clinical therapies.

## 6. Bone Targeted Therapies for Prevention of Skeletal Related Events (SREs)

Metastatic bone disease causes the production of less robust bone mass and thereby leads to fractures and other skeletal related events (SREs) such as spinal cord compression, radiation to bone, or bone surgery. Several studies have investigated the incidence of SREs in patients with advanced prostate cancer. A study using data from two large US health systems reported a cumulative incidence of 41.9% of SREs within 2 years after diagnosis of metastatic PC [32]. Even in patients receiving antiresorptive therapy, 15–20% develop fractures within 2-3 years [33]. Therefore, targeting osteoclast activity in patients with bone metastases from prostate cancer has been demonstrated to be of utmost importance in modern treatment concepts for patients with prostate cancer.

### 6.1. Bisphosphonates

The approval of zoledronic acid for the prevention of SREs in 2002 has significantly contributed to an improved care of patients with PC and bone metastases. In the phase III trial leading to the approval of the drug, 643 patients with castration resistant prostate cancer and osseous metastases were randomized either to 4 mg or 8 mg intravenous zoledronic acid or placebo every four weeks [33]. Due to increased rates of renal toxicity the dosage of the drug in the 8 mg/4 weeks group was reduced to 4 mg (Table 1). Compared to placebo, patients receiving 4 mg zoledronic acid had a significantly lower rate of SREs (38% versus 49%) and a significantly longer time to the first on-study SRE (428 versus 321 days). Since then, zoledronic acid has been standard of care for treatment of bone metastases in prostate cancer for more than one decade. Other bisphosphonates have never played a significant role in the treatment of metastatic bone disease secondary to prostate cancer. Intravenous pamidronate failed to demonstrate a significant efficacy for bone pain and SRE prevention in two-phase III trials [34]. Clodronate, an oral bisphosphonate, has been shown to significantly reduce bone metastases related pain in clinical trials but has never played an important role due to the lack of evidence for SRE prevention [35]. Interestingly, clodronate is the only bisphosphonate, which has been proven to improve the overall survival (OS) of patients with prostate cancer. In a long-term survival follow-up analysis of the PR05 trial, the estimated 10-year overall survival rate was 9% with placebo and 17% with clodronate [36]. For zoledronic acid, a significant improvement of progression-free survival and overall survival has not been observed. The reason, why the delay of SREs does not results in improved survival, is unclear. A couple of preclinical studies have shown direct anticancer effects of bisphosphonates on PC cells [37] and indicate an important role of the mevalonate pathway in the biology of PC [38]. Moreover, studies showing that the mortality of men with hip fractures is as high as 38% within one year after fracture raise expectations that SRE prevention may result in improved survival [39].

### 6.2. Denosumab

The use of zoledronic acid as standard-of-care in patients with castration resistant metastatic prostate cancer has been challenged by the approval of the RANKL inhibitor denosumab. In the phase III trial comparing denosumab with zoledronic acid for SRE-prevention, 1904 men were randomized either to 4 mg zoledronic acid/4 weeks or 120 mg denosumab/4 weeks. The time to first SRE, which was the primary endpoint of the study, was significantly improved in the denosumab arm (20.7 months versus 17.1 months) [40]. Of note, denosumab showed superiority also with regard to pain improvement and pain interference [41]. Recently, symptomatic skeletal related events (SSEs) have been introduced as an endpoint in studies including patients with bone metastases. The phase III trial on Radium 223 in patients with CRPC was the first incorporating SSEs as an endpoint [42]. In contrast to conventional SREs, a nonsymptomatic fracture is not considered as an event when using the endpoint symptomatic SREs. The relevance of nonsymptomatic fractures as an endpoint in clinical trials has been critically discussed [43]. Denosumab has been shown to reduce the risk of SSEs compared to zoledronic acid in patients with CRPC in a reassessment of the data of the phase 3 trial [44].

One of the main advantages of denosumab compared to zoledronic acid is its subcutaneous mode of administration. Compared to zoledronic acid, which shows a rapid decline of serum concentrations after the end-of-infusion, denosumab achieves maximal serum concentrations 5–21 days after subcutaneous injection [45]. These differences in pharmacokinetics are assumed to contribute to differences in efficacy and suppression of bone turnover markers [46]. A dose adjustment due to renal insufficiency is not necessary. Acute phase reactions are less frequent with denosumab. However, the incidence of one of the most concerning side effects of zoledronic acid, osteonecrosis of the jaw, was the same in patients treated with denosumab. Another concerning side effect of antiresorptive treatment, hypocalcemia, was observed more frequently in patients treated with denosumab. Therefore, calcium and vitamin-D supplementation are strongly recommended even prior to treatment with denosumab and calcium serum levels should be checked regularly and supplemented as needed.

### 6.3. SRE Prevention in Castration Sensitive PC

Although current urologic guidelines clearly state that both zoledronic acid and denosumab are recommended only in patients with castration-resistant prostate cancer (CRPC) and bone metastases, the FDA approval is stating more generally that these drugs are indicated in patients with bone metastases from solid tumors. The phase III trials leading to approval of both drugs were performed exclusively in patients with CRPC. Until recently, the efficacy of approved antiresorptive agents in patients with hormone-sensitive PC and bone metastases was unclear. The PR05 trial aiming to assess the efficacy of oral clodronate in patients with metastatic hormone-sensitive PC failed to show significant benefits with regard to bone progression-free survival [47]. However, the long-term follow-up results showed a significant improvement of OS [36]. Most recently, the results of the phase III CALGB 90202 trial including 645 patients with hormone-sensitive prostate cancer were published [48]. Patients were randomized to placebo or 4 mg zoledronic acid every 4 weeks. Surprisingly, the administration of zoledronic acid did not result in a longer time to first SRE (31.9 months for zoledronic acid versus 29.8 months for placebo). Although the study supports recommendations clearly differentiating between castration-sensitive and castration-resistant PC, some aspects have to be discussed critically. The study does not provide any baseline or follow-up BMD values of patients included. As all patients were treated with androgen-deprivation therapy, they have a significantly increased risk of loss of BMD. The combination of decreased BMD and presence of bone metastases are assumed to synergistically impair bone stability. Therefore, information on SRE-related outcome of patients with decreased BMD at study entrance or follow-up investigations is urgently needed to evaluate whether subgroups of patients exist that have a benefit from zoledronic acid in the castration-sensitive stage of disease.

## 7. Osteoclast-Targeting Agents for the Prevention of Bone Metastases

**Figure 1:**
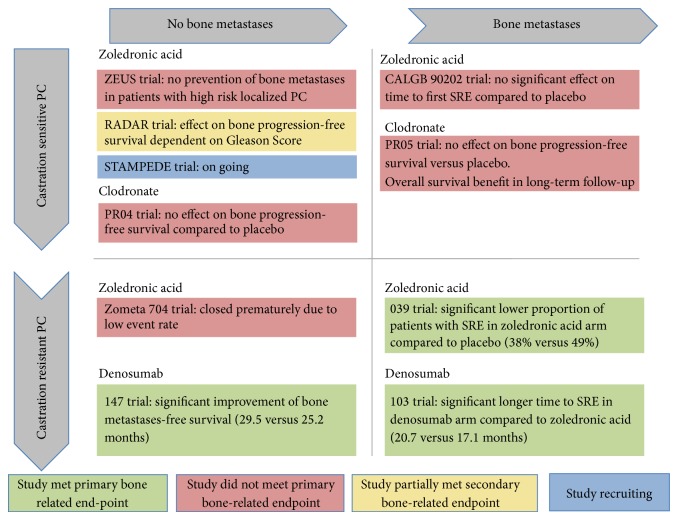
Summary of phase III trials investigating the use of antiresorptive drugs in the context of castration sensitive and castration resistant prostate cancer (PC) with and without bone metastases. SRE: skeletal related event.

Activation of osteoclasts has been shown to be one of the major processes involved in the development of bone metastases from PC [49, 50]. In preclinical studies, the inhibition of osteoclast activation leads to decreased formation of bone metastases [51]. These observations have raised the hope that the administration of antiresorptive drugs may prevent or delay the development of bone metastases and improve oncologic outcome of PC patients. Several clinical trials have been performed to address this issue using bisphosphonates and denosumab in both patients with castration-sensitive and castration resistant PC (Figure 1). Oral clodronate failed to improve bone-metastasis-free survival and overall survival in the PR04 trial [52], which included patients with nonmetastatic PC with features indicating an increased risk of bone metastases. The Zometa 704 trial was closed prematurely due to the low event rate after inclusion of 398 patients with nonmetastatic CRPC [53]. Planned accrual was 991 patients. Recently, the results of the Europen Zometa Study (ZEUS) have been published. In this study, 1433 patients with high-risk localized PC were randomized either to standard of care therapy + 4 mg i.v. zoledronic acid every three weeks for ≤4 years or standard of care therapy alone. After a median follow-up of 4.8 years, no significant improvement in bone metastasis-free survival was observed [54]. The design of the trial including a relatively heterogenous population of patients and the exposure of patients with a relatively low risk of developing bone metastases to treatment with zoledronic acid has been discussed controversially [55, 56]. In the recently published RADAR trial, patients with nonmetastatic PC were randomly allocated to receive either radiation therapy plus short term ADT (6 months) with or without zoledronic acid or radiation therapy plus 18 months of ADT with or without zoledronic acid [57]. In general, no clear benefit for the arms including zoledronic acid was observed. A trend towards improved bone progression-free survival by adding zoledronic acid was only observed for patients with a Gleason Score >7. Interestingly, in patients with a Gleason Score ≤7, the addition of zoledronic acid to ADT resulted in a worse bone progression-free survival. The authors provided the hypothesis that, dependent on the differentiation of the tumor, zoledronic acid exhibits different effects on the tumor cells. Well-differentiated tumor cells might be protected from ADT by zoledronic acid induced changes of interactions of osteoblasts and osteoclasts. To date there is no preclinical data clearly supporting this theory.

In contrast to bisphosphonates, denosumab has provided a proof-of-concept by delaying the onset of bone metastases in patients with CRPC in a clinical phase III trial [58]. In this trial, 1432 patients with castration resistant prostate cancer and no evidence of metastatic bone disease were randomized to receive either 120 mg denosumab every four weeks or placebo. The median time to bone metastases was 29.5 months in the denosumab arm versus 25.2 months in the placebo arm (Hazard Ratio 0.85, 95% CI 0.73–0.98, *P* = 0.028). Further subgroup analysis showed that treatment effects with regard to metastasis prevention are most obvious in patients with a short doubling time of the serum prostate-specific-antigen (PSA) [59]. Overall survival was not affected by the application of denosumab. However, the drug has not been approved for prevention of bone metastases. One reason for that might be the relatively high cumulative rate of ONJs observed within the study (5%).

The future of bone metastases prevention using antiresorptive drugs remains unclear. Although denosumab has provided a proof-of-principle that targeting osteoclasts can inhibit the formation of bone metastases, the clinical significance of the observed median delay of 4.3 months is to be critically discussed [55, 56]. To date, no evidence is available showing that delaying bone metastases delays SREs. Such data would further promote discussions whether the treatment related benefits overweight the relatively high risk of ONJ when using denosumab 120 mg every four weeks, which occurred in 5% of patients. There is a need for further prevention trials with modified study designs. To date it is unclear whether 60 mg of denosumab semiannually might provide a similar metastasis-preventing effect compared to monthly doses of 120 mg. No data have been published so far on the incidence of bone metastases in the long term follow-up of the HALT 138 trial, which was primarily designed to assess the effects on denosumab on CTIBL and CTIBL-associated fractures [11]. The use of denosumab in patients with hormone-sensitive prostate cancer who receive ADT simultaneously might kill two birds with one stone by reducing the risk of CTIBL-induced fractures and preventing/delaying the development of metastases. Recently, preclinical evidence has been provided that castration promotes dissemination of prostate cancer cells into the bone via osteoclast-dependent mechanisms [60]. If this effect is also valid for humans remains to be elucidated. For sure, the optimal timing of treatment will remain one of the major challenges for future trials investigating bone metastasis prevention by antiresorptive drugs.

## 8. Effect of Antineoplastic Agents on Bone Complications

Recent phase III trials in patients with castration resistant prostate cancer have shown that cytochrome P (CYP17) inhibitors and androgen receptor inhibitors not only result in a significant improvement of overall survival but also have a positive effect on the incidence of symptomatic skeletal related events [61, 62]. Abiraterone and enzalutamide have been recently approved by the FDA and the European Medicines Agency (EMA) for the treatment of patients with CRPC. To date it is unclear whether they delay SREs only by their antitumor effect or if they also have an effect on the interaction of tumor cells with the bone. A potential synergistic effect of these drugs with antiresorptive drugs has not been demonstrated yet. The alpha-emitter radium-223-chloride specifically targets osteoblastic bone metastases and achieved an improvement of the median overall survival by 2.8 months in a large phase III trial [42]. It has been also demonstrated that the application of Radium 223 results in a significant delay of the first symptomatic skeletal related events [63]. Concerns where antiresorptive drugs might inhibit the uptake of Radium-223-chloride and impact drug efficacy were countered by the finding that the use of antiresorptive agents had no negative impact on outcome in a subanalysis of the trial [64]. The c-met-inhibitor cabozantinib, which has been demonstrated to have considerable effects on bone metastases in the phase II trial with a complete resolution of metastases in bone scans in 12% of patients failed to provide significant benefits on overall survival in the recently closed phase III trial [65, 66]. The 17,20-lyase inhibitor orteronel (TAK-700) also failed to demonstrate a significant benefit on overall survival in two-phase III trials including chemotherapy naïve patients and patients with CRPC that had progressed during or following docetaxel [67, 68]. Although in both trials a significant improvement of radiographic progression-free survival has been observed, approval of these drugs in patients with CRPC is not expected. Data on skeletal related events in these trials have not been published so far.

## 9. Conclusions

Antiresorptive drugs are the standard of care in the treatment of patients with advanced prostate cancer. For both the treatment of CTIBL and the prevention of SREs antiresorptive drugs have demonstrated to provide significant benefit for PC patients. Whereas zoledronic acid has been the standard of care for more than a decade for prevention of SREs, denosumab is currently used as an antiresorptive agent for a high proportion of patients who are newly diagnosed with metastatic bone disease. The use of osteoclast targeting drugs for the prevention of bone metastases is feasible but not recommended by current guidelines due to significant side effects and questionable oncologic benefit. More trials are needed to verify potential populations that may profit from the adjuvant use of these drugs. The approval of new antineoplastic agents for CRPC that have a positive effect on the incidence of SREs requires further evaluation of potential interactions and synergistic effects with antiresorptive drugs.

## Figures and Tables

**Table 1:** Characteristics of antiresorptive agents used for patients with prostate cancer.

	Bisphosphonates	Denosumab
Target	Osteoclast	Osteoclast
Mechanism of action	Inhibition of mevalonate pathway	AB against RANKL
Route of administration	Intravenous, oral	Subcutaneous
Contraindication	Renal insufficiency	Hypocalcaemia
Adverse events	Osteonecrosis of the jaw, acute phase reaction Gastrointestinal (for oral BPs)	Osteonecrosis of the jaw, hypocalcaemia

AB: antibody.
